# A case of right renal cell carcinoma with an inferior vena cava tumor thrombus extending above the diaphragm resected without cardiopulmonary bypass

**DOI:** 10.1016/j.eucr.2025.102982

**Published:** 2025-02-10

**Authors:** Haruto Honda, Norichika Ueda, Kentaro Takezawa, Taigo Kato, Koji Hatano, Shinichiro Fukuhara, Norio Nonomura, Atsunari Kawashima

**Affiliations:** Department of Urology, Osaka University Graduate School of Medicine, 2-2 Yamadaoka, Suita, Osaka, 565-0871, Japan

**Keywords:** Renal cell carcinoma, Tumor thrombus, Cardiopulmonary bypass, Tumor thrombectomy, Ultrasound

## Abstract

We preoperatively evaluated the tumor thrombus of right renal cell carcinoma cT3cN0M0 extending close to the right atrium using abdominal ultrasound. We found that invasion of the inferior vena cava (IVC) by the tumor thrombus was limited to the caudal side of the hepatic vein. We clamped the caudal IVC, left renal, and hepatic veins but not the cranial IVC. Incising the IVC in this situation caused retrograde flow, moving the floating tumor thrombus caudally. This enabled rapid extraction of the tumor thrombus and cranial IVC clamping below the hepatic vein. Consequently, tumor thrombectomy was successfully performed without cardiopulmonary bypass.

## Highlights

• Information on tumor thrombus dynamics can be obtained by ultrasonography.
• Backflow of blood in IVC moves the floating tumor thrombus caudally.
• After tumor thrombus removal, the cranial IVC can be clamped at a more caudal site.
• Some Level IV tumor thrombus can be resected without cardiopulmonary bypass.

## 1 Introduction

Despite rapid advances in pharmacological therapy, nephrectomy and tumor thrombectomy are the most effective treatments for non-metastatic renal cell carcinoma (RCC) with a tumor thrombus extending into the inferior vena cava (IVC).^1^ In cases where the tumor thrombus extends cranially beyond the diaphragm, cardiopulmonary bypass is typically required to ensure optimal surgical exposure and bleeding control. However, the use of cardiopulmonary bypass carries a high risk of severe complications and is highly invasive.^1^ Herein, we report a case of renal cancer with a tumor thrombus extending near the right atrium, which was successfully resected without cardiopulmonary bypass. At the start of the IVC incision, the cranial IVC was not clamped, but the caudally directed blood flow within the IVC moved the floating tumor thrombus caudally. We were able to extract the tumor thrombus from the IVC swiftly and clamp the cranial IVC caudal to the hepatic vein. As a result, we could perform tumor thrombectomy safely without the use of cardiopulmonary bypass.

## 2 Case presentation

**Fig. 1:**
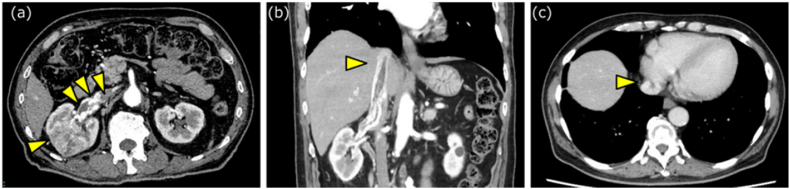
Contrast-enhanced computed tomography (a) Tumor with heterogeneous enhancement in the early contrast phase (arrow) and tumor thrombus extending from the right renal vein into the inferior vena cava (triangle). (b–c) The tip of the tumor thrombus extends beyond the diaphragm, approaching the vicinity of the right atrium.

A 72-year-old man presented to our hospital with a complaint of gross hematuria. He had hypertension and diabetes. There was no other past medical history. Abdominal ultrasonography (US) revealed a mass in the right kidney. Abdominal contrast-enhanced computed tomography (CT) revealed a mass measuring 44 mm in the right kidney that showed heterogeneous enhancement in the early phase and a washout effect in the delayed phase. In addition, a tumor thrombus extending from the right renal vein to the IVC was observed, whose tip was in proximity to the right atrium (Fig. 1a–c). No metastasis was detected. The patient was diagnosed with right RCC (cT3cN0M0). His general condition was good, and he had no symptoms other than mild gross hematuria. After consulting the patient and his family, we decided to perform radical right nephrectomy and tumor thrombectomy. We initiated anticoagulation therapy to prevent thrombus formation on the patient's tumor thrombus.

**Fig. 2:**
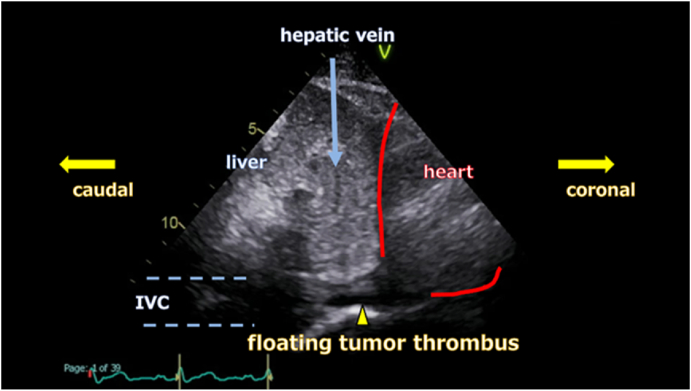
Abdominal ultrasonography The IVC region invaded by the tumor thrombus (yellow triangle) is located below the hepatic vein (blue arrow). The tumor thrombus above the hepatic vein is mobile and floating, while the IVC site where the tumor thrombus has invaded is nearly occluded, resulting in very low blood flow. IVC; inferior vena cava.

Preoperatively, we evaluated the tumor thrombus using US. The thrombus extended continuously to the vicinity of the right atrium; however, the area suspected of having IVC wall invasion was caudal to the hepatic vein. At this site, the tumor occupied the IVC, and blood flow was poor. In contrast, the thrombus cranial to the occupied segment was floating, highly mobile, and surrounded by abundant blood flow (Fig. 2). Considering the dynamic assessment of the tumor thrombus and surrounding hemodynamics, we concluded that surgery could be performed safely without the use of cardiopulmonary bypass.

**Fig. 3:**
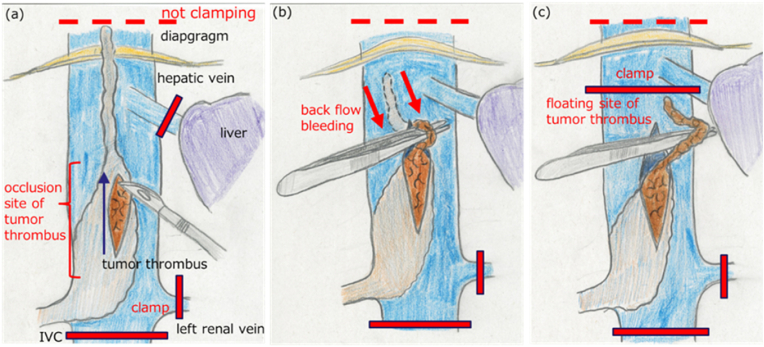
Tumor thrombus removal (a) The IVC was clamped below the tumor thrombus, as well as at the contralateral renal vein and hepatic vein, before incising the IVC without clamping it above the tumor thrombus (red bar). (b) Backflow bleeding (arrow) from cranial to caudal occurred at the incision site. Using this backflow of blood, we were able to quickly and easily extract the floating tumor thrombus from the IVC. (c) We were able to clamp the IVC below the hepatic vein sooner and release the clamp on the hepatic vein in a short time. IVC; inferior vena cava.

**Fig. 4:**
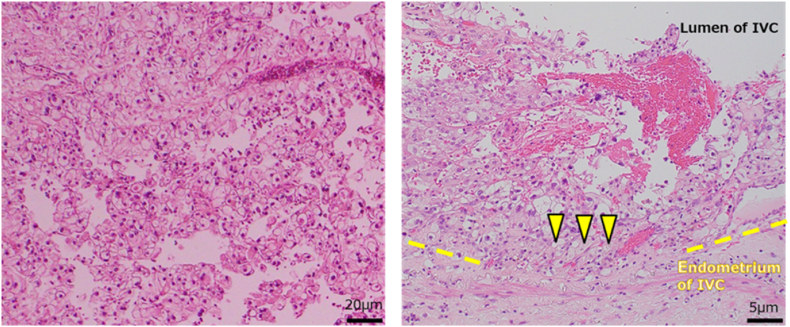
Histopathological findings The histopathological diagnosis was grade III clear-cell renal cell carcinoma. The tumor thrombus has invaded the IVC walls (triangle), and the surgical margins are negative. IVC; inferior vena cava.

To prioritize safety, we initiated surgery after placing venous drainage catheters in the femoral and jugular veins so that we could switch to cardiopulmonary bypass as soon as possible, if necessary. Intraoperative US revealed no changes from the preoperative assessment. Following ligation and cutting of the right renal artery and completion of dissection around the right kidney, we attempted to interrupt the IVC cranial to the tumor thrombus from the abdominal cavity. However, no regression of the tumor thrombus was observed after renal artery ligation. Therefore, it was not possible to manually move the tip of the tumor thrombus caudally, and this proved difficult even after diaphragmatic incision. Conventionally, this would have been the time to switch to cardiopulmonary bypass, but per our preoperative plan, we blocked the IVC caudal to the tumor, left renal vein, and hepatic veins, without cranial interruption, and commenced IVC incision. We started the incision in the thrombus-filled segment devoid of wall infiltration and progressed cranially without compromising the tumor. This segment exhibited poor blood flow, resulting in minimal bleeding. Advancing the incision cranially past the thrombus-filled segment to the floating segment increased bleeding. Then, the tip of the thrombus moved caudally with retrograde flow, facilitating swift and easy extraction of the floating thrombus segment via forceps manipulation. Consequently, after removing the tumor thrombus, we were able to clamp the IVC on the caudal side of the hepatic vein, and then, the hepatic vein could be declamped. (Fig. 3a–c). Subsequently, good hemostasis was achieved, and we could resect the wall of the IVC infiltrated by the tumor. We removed the right kidney and tumor thrombus en bloc. We repaired the IVC using a pericardial patch, and surgery was completed. The operative time was 8 h and 3 min, and the volume of blood loss was 2370 mL. The total hepatic vein occlusion time was 7 min and 30 s. No abnormalities were detected during intraoperative transesophageal ultrasound monitoring of the tumor thrombus or pulmonary embolus. As the defect in the IVC was reconstructed using a bovine pericardial patch, anticoagulation therapy was resumed intraoperatively to prevent thrombus formation and was continued permanently postoperatively. The pathological diagnosis was grade III clear-cell RCC, with complete resection of the vascular wall infiltration and negative surgical margins (Fig. 4). The patient's postoperative course was uneventful, and he was discharged without complications on postoperative day 21.

## 3 Discussion

In this case, the tumor thrombus of the RCC in the right kidney extended to the vicinity of the right atrium, and we were not able to clamp the IVC cranial to the thrombus at the start of the operation. However, by utilizing the reverse blood flow toward the caudal direction in the IVC, we were able to quickly and safely extract the floating tumor thrombus from the IVC and clamp the head of the IVC caudal to the hepatic vein. As a result, we were able to safely perform nephrectomy and tumor thrombectomy without using cardiopulmonary bypass, thereby reducing the invasiveness of the operation.

RCC has a propensity for venous invasion, often extending into the renal vein and the IVC, and, in rare cases, crossing the diaphragm to reach the right atrium.^2^ The incidence of RCC involving the IVC is estimated to be 4–10 %.^3^ Despite significant advancements in pharmacological therapy for RCC, their efficacy in managing tumor thrombus remains uncertain 4. Consequently, nephrectomy and tumor thrombectomy remain the cornerstone treatments for RCC with tumor thrombus in the absence of metastasis.

Accurate preoperative assessment of the height of the tumor thrombus is crucial because it affects the operative procedure, operative time, blood loss, and risk of complications.^5^^,^^6^ A tumor thrombus below the diaphragm can usually be removed by clamping the IVC cranial to the thrombus from the abdominal cavity. In contrast, a tumor thrombus above the diaphragm is usually removed with cardiopulmonary bypass.^1^^,^^7^^,^^8^ The benefits of cardiopulmonary bypass include ease of bleeding control, stabilized hemodynamics, cardiac access through sternotomy, and prevention of pulmonary embolism during thrombus extraction.^1^ However, the disadvantages include increased surgical invasiveness, high intraoperative and postoperative bleeding due to systemic heparinization, and potential tumor cell dissemination via the cardiopulmonary bypass circuit.^9^^,^^10^

We aimed to avoid cardiopulmonary bypass due to the attendant challenges. In this case, although the cranial end of the tumor thrombus extended beyond the diaphragm, its invasion into the IVC wall was confined below the hepatic vein, and the portion of the thrombus above the hepatic vein was free-floating. We anticipated that we would be able to retract the floating thrombus below the hepatic vein. Furthermore, we hypothesized that by clamping the caudal IVC, left renal vein, and hepatic veins while leaving the cranial IVC unclamped, retrograde blood flow within the IVC would push the floating thrombus downward. This would allow for easy and rapid removal of the tumor thrombus. After removing the thrombus, we expected to clamp the IVC below the hepatic vein, minimizing the duration of hepatic vein occlusion. Ultimately, not only were we able to avoid cardiopulmonary bypass, but we also significantly reduced the hepatic vein occlusion time using this surgical approach.

When comparing this case to those of level IV renal cancer surgeries involving cardiopulmonary bypass conducted at our hospital between 2008 and 2022, this case demonstrated better outcomes in terms of operative time, blood loss, hepatic vein clamping duration, and hospital stay, resulting in a less invasive procedure overall (Table 1). At present, there are no large-scale studies on level IV renal cancer surgeries, making it difficult to compare progression-free survival and overall survival (OS) across different surgical techniques. However, the invasiveness of the surgery could significantly affect the timing of postoperative treatments, which might, in turn, influence OS. Therefore, we believe that it is crucial to carefully evaluate whether cardiopulmonary bypass can be avoided in each case.

**Table 1:** Comparison of the current case's data with those of cases treated with cardiopulmonary bypass for renal cell carcinoma with level IV tumor thrombus between 2008 and 2022 at our hospital.

Case	C-stage	Age	Sex	Use of cardiopulmonary bypass	Operative time	Blood loss volume	Hepatic vein interception time	Length of hospital stay
2009	cT3cN0M0	65	Male	Yes	11 hours and 5 minutes	5110 mL	12 minutes and 40 seconds	36 days
2012	cT3cN0M0	57	Female	Yes	16 hours and 21 minutes	7470 mL	9 minutes	115 days
2014	cT3cN0M0	73	Male	Yes	10 hours and 50 minutes	3600 mL	25 minutes	22 days
2016	cT3cN0M0	72	Male	Yes	12 hours and 53 minutes	26,400 mL	28 minutes	47 days
2022 (This case)	cT3cN0M0	72	Male	No	8 hours and 3 minutes	2370 mL	7 minutes and 30 seconds	21 days

The key factor enabling this surgical approach was that thrombus infiltration into the IVC wall and its obstruction were limited to areas below the hepatic veins. Assessing the extent of the thrombus required not only CT or magnetic resonance imaging but also dynamic ultrasound imaging to evaluate characteristics such as wall infiltration, mobility, and IVC blockage. Preoperative ultrasound assessment of the tumor thrombus was highly valuable in determining both the feasibility of removing the thrombus from the IVC and the ideal site for its extraction. While not applicable to all cases, this approach can greatly minimize surgical invasiveness for level IV renal cancer when conditions are favorable. Reducing invasiveness translates to fewer complications during and after surgery, and also allows patients to start additional treatments more quickly if needed. Therefore, this method should be considered when the situation is suitable.

## 4 Conclusion

By evaluating tumor thrombus dynamics with ultrasound imaging, we were able to safely perform surgery without cardiopulmonary bypass for a level IV RCC tumor thrombus. Although this approach is not universally applicable, it is significantly less invasive when the conditions are favorable and should be considered an option in surgical strategies.

## Glossary

Pericardial patch: It is processed from bovine pericardium and used as a pericardial substitute and for vascular repair and septal defect repair.

## CRediT authorship contribution statement

**Haruto Honda:** Writing – original draft. **Norichika Ueda:** Writing – original draft. **Kentaro Takezawa:** Supervision. **Taigo Kato:** Supervision. **Koji Hatano:** Supervision. **Shinichiro Fukuhara:** Supervision. **Norio Nonomura:** Supervision, Project administration. **Atsunari Kawashima:** Writing – review & editing, Supervision, Project administration, Conceptualization.

## Informed consent

The patient provided written permission for the publication of this case report.

## Data statement

Raw data were generated at Osaka University. Derived data supporting the findings of this study are available from the corresponding author on request.

## Funding

This study did not receive any specific grants from funding agencies in the public, commercial, or non-profit sectors.

## Conflicts of interest

The authors declare no conflicts of interest.
